# Improving a recombinant *Zymomonas mobilis* strain 8b through continuous adaptation on dilute acid pretreated corn stover hydrolysate

**DOI:** 10.1186/s13068-015-0233-z

**Published:** 2015-03-31

**Authors:** Ali Mohagheghi, Jeffrey G Linger, Shihui Yang, Holly Smith, Nancy Dowe, Min Zhang, Philip T Pienkos

**Affiliations:** National Renewable Energy Laboratory, National Bioenergy Center, 15013, Denver West Parkway, Golden, CO 80401 USA

**Keywords:** *Zymomonas mobilis*, Xylose, Pretreated corn stover, Adaptation, Turbidostat, Next-generation sequencing (NGS)

## Abstract

**Background:**

Complete conversion of the major sugars of biomass including both the C_5_ and C_6_ sugars is critical for biofuel production processes. Several inhibitory compounds like acetate, hydroxymethylfurfural (HMF), and furfural are produced from the biomass pretreatment process leading to ‘hydrolysate toxicity,’ a major problem for microorganisms to achieve complete sugar utilization. Therefore, development of more robust microorganisms to utilize the sugars released from biomass under toxic environment is critical. In this study, we use continuous culture methodologies to evolve and adapt the ethanologenic bacterium *Zymomonas mobilis* to improve its ethanol productivity using corn stover hydrolysate.

**Results:**

A turbidostat was used to adapt the *Z. mobilis* strain 8b in the pretreated corn stover liquor. The adaptation was initiated using pure sugar (glucose and xylose) followed by feeding neutralized liquor at different dilution rates. Once the turbidostat reached 60% liquor content, the cells began washing out and the adaptation was stopped. Several ‘sub-strains’ were isolated, and one of them, SS3 (sub-strain 3), had 59% higher xylose utilization than the parent strain 8b when evaluated on 55% neutralized PCS (pretreated corn stover) liquor. Using saccharified PCS slurry generated by enzymatic hydrolysis from 25% solids loading, SS3 generated an ethanol yield of 75.5% compared to 64% for parent strain 8b. Furthermore, the total xylose utilization was 57.7% for SS3 *versus* 27.4% for strain 8b. To determine the underlying genotypes in these new sub-strains, we conducted genomic resequencing and identified numerous single-nucleotide mutations (SNPs) that had arisen in SS3. We further performed quantitative reverse transcription PCR (qRT-PCR) on genes potentially affected by these SNPs and identified significant down-regulation of two genes, ZMO0153 and ZMO0776, in SS3 suggesting potential genetic mechanisms behind SS3’s improved performance.

**Conclusion:**

We have adapted/evolved *Z. mobilis* strain 8b for enhanced tolerance to the toxic compounds present in corn stover hydrolysates. The adapted strain SS3 has higher xylose utilization rate and produce more ethanol than the parent strain. We have identified transcriptional changes which may be responsible for these phenotypes, providing foundations for future research directions in improving *Z. mobilis* as biocatalysts for the production of ethanol or other fuel precursors.

## Background

The economical production of cellulosic ethanol requires optimal conversion of the major sugar components of biomass including both the C_5_ and C_6_ sugars. Accordingly, the chosen ethanologen must be either natively competent to use these sugars or these traits must be engineered into the organism. An example of the latter case is the recombinant *Zymomonas mobilis* strain 8b, which was genetically modified to co-ferment glucose and xylose to ethanol [1]. While the implementation of heterologous carbon assimilation pathways can instill the basal ability to utilize new sugars, the efficiency of these new metabolisms is often low, and this effect can be exacerbated in the presence of lignocellulosic hydrolysate [2]. The biomass pretreatment process, necessary for producing fermentable sugars, produces compounds that are frequently inhibitory to microorganisms [3,4]. Specifically, dilute-acid pretreatment produces high levels of xylose and digestible cellulose, but it also produces toxic compounds such as acetic acid and the sugar degradation products, furfural and hydroxymethylfurfural (HMF) [2]. Additionally, high salt concentrations develop from the sulfuric acid pretreatment and the ammonium hydroxide neutralization steps. The combination of inhibitors, salts, mixed sugars, and ethanol produced from the fermentation (generally referred to as ‘hydrolysate toxicity’) is a huge burden on the microorganism that prevents complete utilization of the sugars and high ethanol production [2]. In the case of our recombinant *Z. mobilis* strains, non-native C_5_ sugar utilization is a particular problem in the presence of inhibitors and ethanol [5].

There exist numerous strategies to circumvent or alleviate the hydrolysate toxicity on the microorganism. Primarily, these involve either modifying the biomass pretreatment process to generate less toxic material or to adapt or engineer the microorganism to tolerate the inhibitory compounds [6-8]. With regard to the former strategy, one route is to use less severe pretreatment conditions, which acts to reduce the salt concentration and sugar degradation products. Additionally, removing acetic acid either before or after pretreatment is another strategy with the potential to improve fermentation [9,10]. However, there are tradeoffs associated with modifying pretreatment or removing acetic acid. Less severe pretreatment conditions usually result in less digestible cellulose so the burden lies with the enzymes to achieve high glucose yield and convert oligomeric C_5_ sugars to monomers [3]. Removing acetate before or after pretreatment adds processing steps that increase the process cost [3].

An alternative strategy to modifying process configurations is to develop a more robust microorganism that is better able to utilize the biomass sugars in the toxic environment of pretreated biomass. Genetic engineering can be a powerful tool for imparting new capabilities in microorganisms. However, robustness is a complex phenotype, and improving robustness can involve many genes or metabolic nodes; all of which can be controlled by undefined regulatory mechanisms. Since targeted engineering of superior strains involves detailed understanding of the phenotype/genotype relationship and can often require high throughput-directed evolution techniques [11] for enhanced enzyme activities, it makes this task daunting and difficult to achieve. However, the classical strategy of adaptation or continuous culture with or without mutagenesis is still powerful because of the following: (1) it is relatively easy to generate microbial populations with high genetic diversity, (2) through fitness selection it is possible to select for superior microorganisms to many kinds of environmental challenge [12], and (3) the advance of next-generation sequencing techniques makes the correlation between genotypic change and phenotypic change feasible [6,13].

Continuous culture techniques, including chemostats and turbidostats, offer a very efficient way of adapting the microorganism to a specific environment [14]. The fermentation conditions can be iteratively altered or increased in severity, and the microorganism’s performance can be assessed as the population adapts to the new environment. This approach requires a great deal of time, appropriate tools, and careful attention to experimental design and technical details to maximize the likelihood that the culture conditions select for the desired phenotype. We applied the time-tested strategy of continuous culture using a turbidostat to continually adapt the microorganism population to neutralized liquor from pretreated corn stover. Given the heightened levels of furfural within hydrolysate and the knowledge that this compound forms DNA-damaging free radicals [4], we speculated that hydrolysate itself is likely to act as a mutagen capable of creating genetic diversity in which our selection strategy could act upon. We isolated several colonies at the end of a 70-day turbidostat run and evaluated their performance and sugar utilization in a whole slurry pretreated corn stover (PCS) fermentation. We generated strains with marked fermentative improvement and accordingly subjected one of these strains to next-generation genomic resequencing. Based on the SNPs observed in the more robust strain, we hypothesize that altered regulation of gene expression is likely to be responsible for the increased performance of our strain. Regardless of the genetic mechanism behind the strain’s enhanced performance, we have isolated a novel *Z. mobilis* strain with enhanced fermentation properties thus validating our turbidostat-based adaptation approach.

## Results and discussion

**Figure 1:**
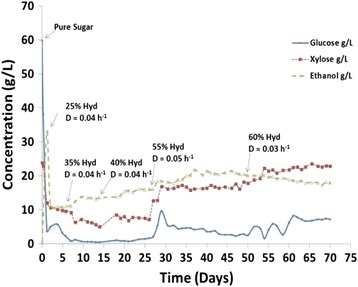
**Continuous fermentation profile during adaptation of** ***Zymomonas mobilis*** **strain 8b on pretreated corn stover hydrolysate.** At pH 5.8, temperature 33°C. Glucose, xylose, and ethanol concentrations are plotted, and points where the concentration of hydrolysate was increased are annotated. ‘D’ represents the dilution rate.

**Figure 2:**
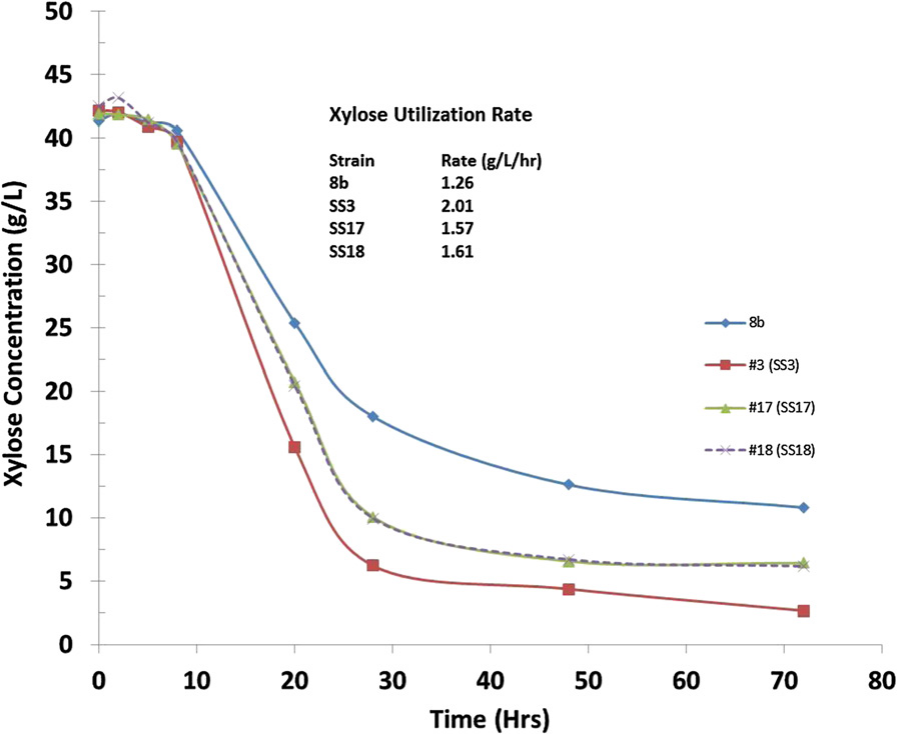
**Comparison of the rate of xylose utilization for sub-strains and parent strain 8b.** Comparison of the rate of xylose utilization for sub-strain SS3, SS17, and SS18 compared to parent strain 8b on 55% neutralized liquor. Xylose consumption profiles and xylose utilization rate are reported for each of four strains examined.

**Figure 3:**
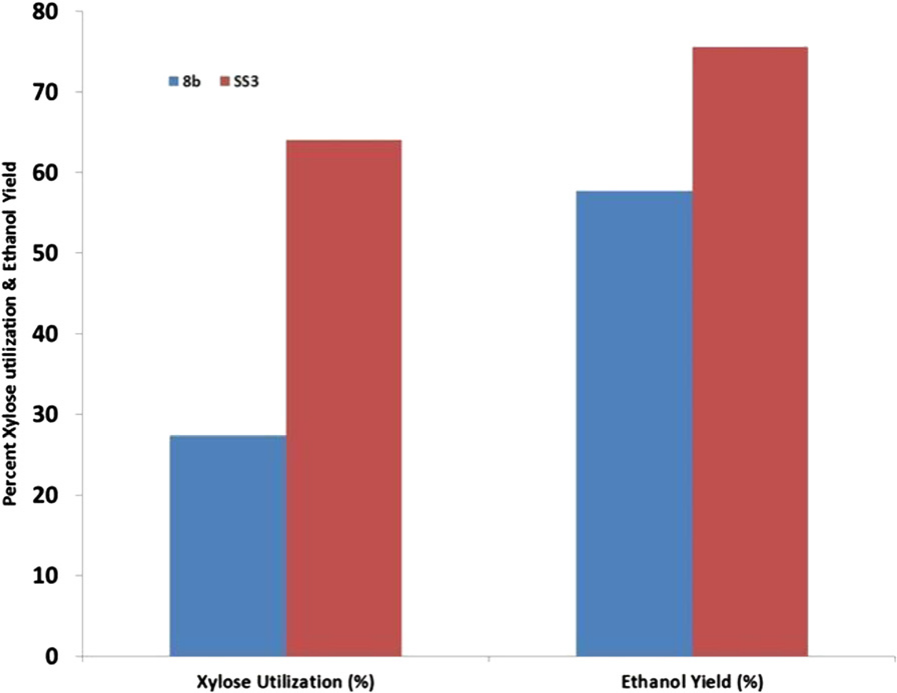
**Comparison of xylose utilization and ethanol yield during fermentation.** Comparison of xylose utilization and ethanol yield during fermentation by two strains (8b and sub-strain SS3) on saccharified, neutralized liquor from 25% solids loading.

We used a turbidostat to adapt the *Z. mobilis* strain 8b in pretreated corn stover liquor. In order to slowly apply selective pressure, the operation was initiated using pure sugar (glucose and xylose) as the sole carbon source in the fermentation. Gradually, neutralized liquor was fed to the fermentor at different dilution rates. The concentration of the liquor was increased as adaptation was progressing. Simultaneously, the dilution rate or feeding rate was lowered to provide more opportunity for the cells to adapt. Cell counts were measured using serial dilution colony counting before increasing hydrolysate concentration to check for viability. After 70 days of operation, the adaptation was stopped due to washout of the cells and the fermentation broth was plated on pure sugar (glucose and xylose) and 30% hydrolysate plates. Figure 1 shows the turbidostat profile for the adaptation process from which 20 colonies were selected. As this figure shows, adaptation was initiated at a 25% neutralized liquor level with a dilution rate of 0.04 h^−1^. The percentage hydrolysate used for dilution was increased stepwise up to a final level of 60%. At this point, the cells began washing out, unable to grow at a pace faster than the dilution rate. We terminated the adaptation at this point and isolated cells from the turbidostat before complete washout. The selected colonies were pre-evaluated for fermentation performance using 55% neutralized hydrolysate in a shake flask and further evaluated in a pH-controlled fermentation leading to the selection of three sub-strains (SS3, SS17, and SS18) for final evaluation. These three sub-strains (SS3, SS17, and SS18) and the parent strain 8b were evaluated on 55% ammonia-neutralized hydrolysate for xylose utilization under pH-controlled conditions. Figure 2 compares the profiles and rate of xylose utilization for these strains. As Figure 2 shows, sub-strain SS3 had a higher xylose utilization rate of 2.01 g/L/h, 59% higher than the parent strain 8b at 1.28 g/L/h. This final evaluation on 55% neutralized hydrolysate led to the selection of SS3 as the best performing strain which had the highest ethanol yields of the three sub-strains. Note that the xylose utilization profiles of SS17 and SS18 overlap almost exactly making them difficult to distinguish on this plot. SS3 was then tested under more realistic process conditions, using sugar produced from enzymatic hydrolysis of the original pretreated hydrolysate diluted to a 25% solids loading that was ‘neutralized’ with ammonium hydroxide under pH-controlled fermentation. As Figure 3 shows, the ethanol yield achieved was 75.5% for the adapted strain SS3, compared to 64% for strain 8b. Xylose utilization for the adapted strain was 57.7% but only 27.4% for strain 8b. Overall, ethanol yield was about 18% higher and xylose utilization doubled for the adapted strain compared to the parent strain. Also, at the end of the fermentation (7 days), the viability of the adapted strain was higher than parent strain 8b. The cell count on the plate of rich media supplemented with glucose (RMG(2%)) was 7 × 10^6^ for SS3 *versus* 5 × 10^3^ for 8b, which demonstrates increased robustness for SS3.

**Figure 4:**
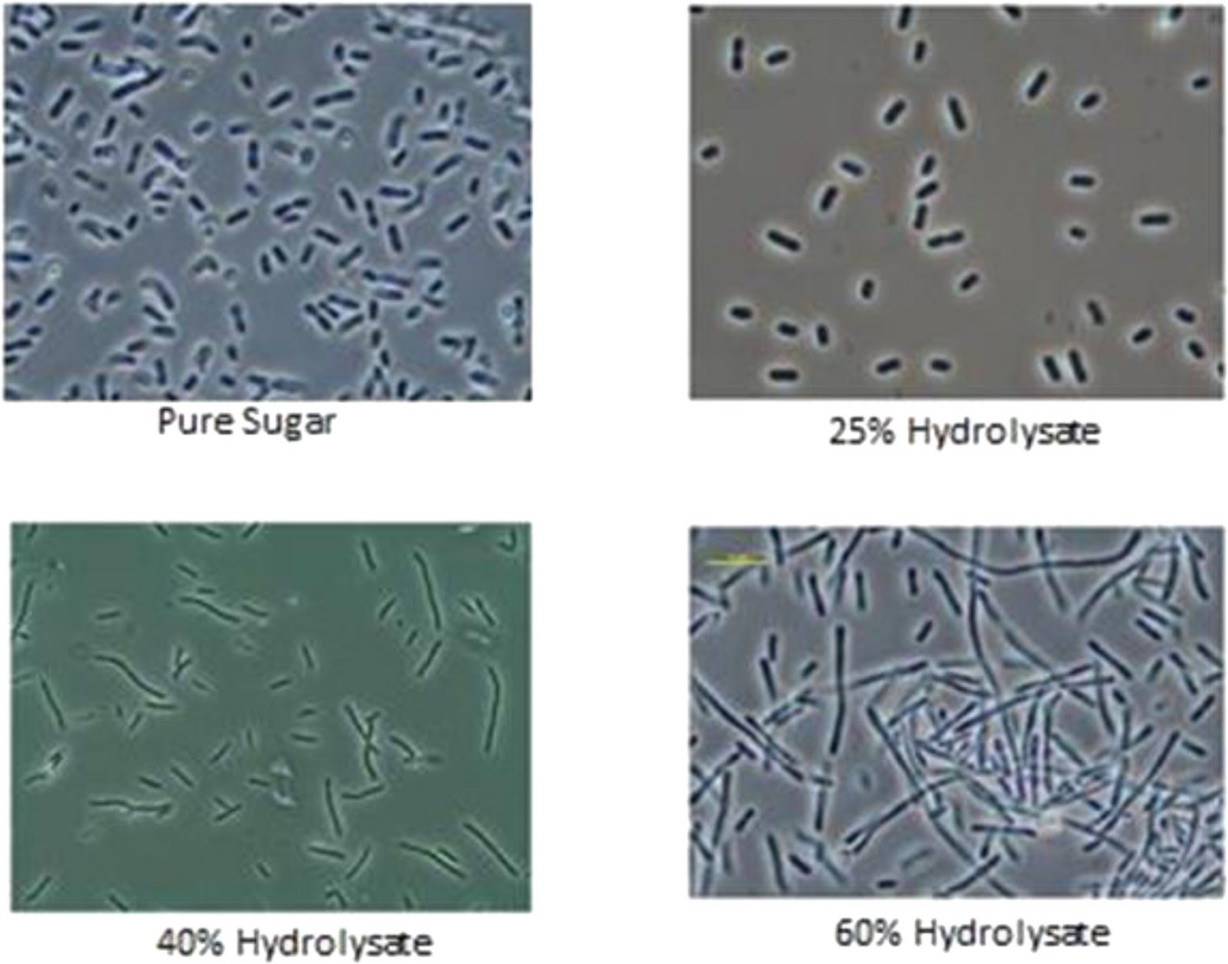
**Morphological changes of strain 8b during adaptation on different hydrolysate concentrations as observed by light microscopy.**

We noted an interesting observation during the fermentations: the morphologies of the *Z. mobilis* strains were drastically altered as the concentration of hydrolysate was increased, as shown by light microscopy (Figure 4). We are unsure of the cause of this phenotype but hypothesize that it is a stress response to one or more of the inhibitory compounds of the hydrolysate. Such morphological disturbances have been described previously in *Z. mobilis* when exposed to compounds frequently present in hydrolysate [4,15]. Therefore, the morphological changes seen in SS3 suggest that despite its improved fermentative performance, it remains physiologically challenged by the toxic hydrolysate environment.

### Identification of genetic changes in SS3

**Figure 5:**
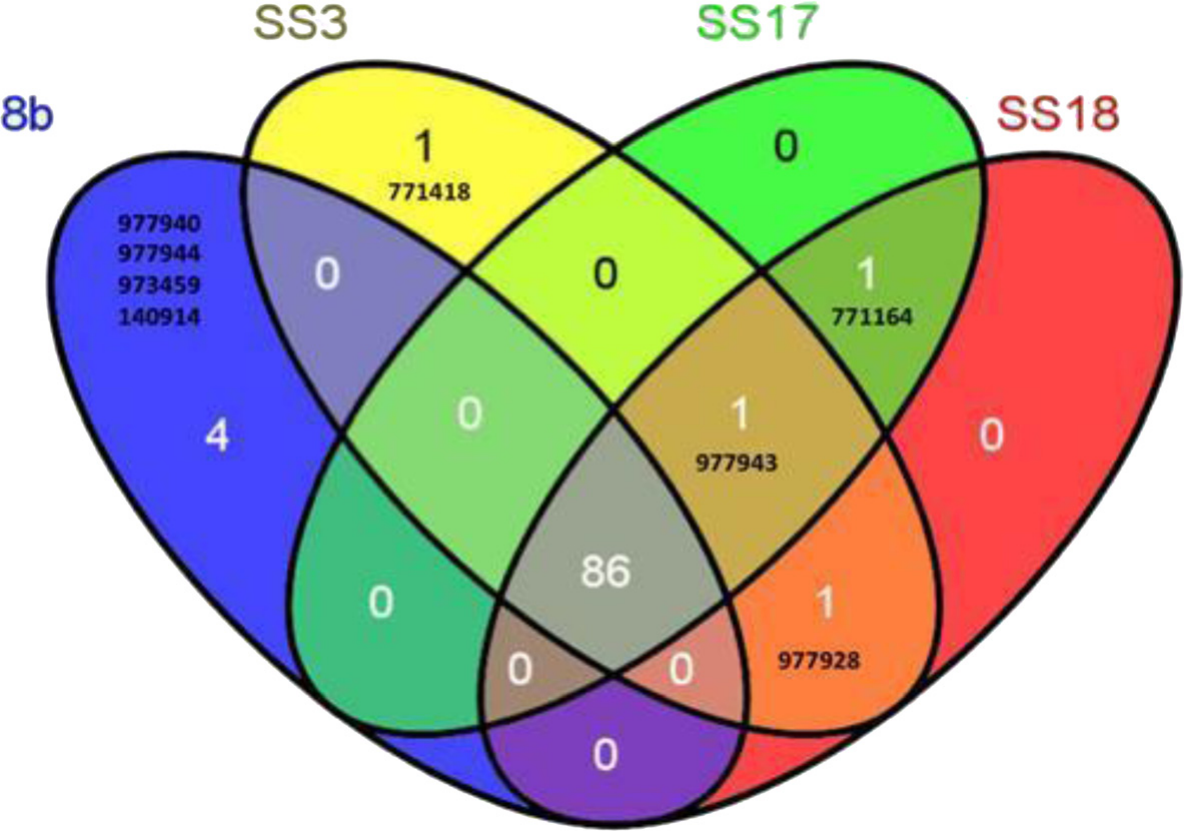
**Single nucleotide polymorphism difference among parental strain 8b and three sub-strains.** The single nucleotide polymorphism (SNP) difference among parental strain 8b and three sub-strains of SS3, SS17, and SS18 depicted as a Venn diagram. The six-digit number indicates the SNP location in the *Z. mobilis* 8b chromosome. Specifically, the SNP locations and associated SNP changes, as well as the genes affected, are: 771418:G- > T, intergenic region between ZMO0774 and ZMO0776; 977943:T- > G, intergenic region between ZMO0959 and ZMO0961; 977928:T- > G, intergenic region between ZMO0959 and ZMO0961; 771164:G- > A, ZMO0774 N-terminal; 977940:G- > T, intergenic region between ZMO0959 and ZMO0961; 977944:-- > G, intergenic region between ZMO0959 and ZMO0961; 973459:T- > A, pseudogene ZMO0955; 140914:CC- > GT, intergenic region between ZMO0152 and ZMO0153.

To determine the underlying genetic determinants responsible for SS3’s enhanced xylose utilization and ethanol productivity, we used next-generation sequencing (NGS) technologies to identify the potential genetic changes in SS3 as well as SS17 and SS18. Parental strain 8b was used as the reference strain for SNP characterization. Seven SNPs were different between 8b and SS3 (Figure 5). SS3 had one SNP in common with both SS17 and SS18 and one in common with only SS18. Intriguingly, nearly all of the SNPs found in SS3 are within the promoter region between two genes. For example, four SNPs are within the promoter region between ZMO0961 and ZMO0959. ZMO0959 encodes a monofunctional biosynthetic peptidoglycan transglycosylase, and ZMO0961 encodes a cytochrome C class I enzyme. Additionally, one SNP is within the promoter region between a LysR-family transcriptional regulator gene ZMO0774 and a putative methyltransferase gene ZMO0776. Interestingly, in a related study, we identified a SNP in ZMO0774 in a *Z. mobilis* strain with enhanced xylose utilization capacity [12]. Yet another SNP is located within the promoter region between ZMO0152 and ZMO0153. The identification of a SNP within a promoter region suggests a strong role for altered transcriptional activities, rather than protein mutations leading to the enhancement of xylose utilization and ethanol productivity.

### Altered transcript levels between 8b and SS3

To determine whether any of the genes affected by SNPs in strain SS3 were altered at a transcriptional level, we performed quantitative real-time reverse transcription PCR (qRT-PCR) on transcripts from the genes adjacent to SNPs and several glycolytic genes. We performed this analysis on strains grown both in RMG and in PCS hydrolysate. However, it is important to note that due to limited supply of the original material, this hydrolysate was different from the hydrolysate used for the original strain adaptation. Notably, the material used to generate the hydrolysate in this gene expression experiment has been de-acetylated prior to dilute-acid pretreatment as has now become our standard procedure owing to superior convertibility [16] and reduced toxicity. Table 1 shows side-by-side comparisons of the composition of these hydrolysates. These values were determined as described previously [17] Notably, the furfural and HMF concentrations are much lower in the de-acetylated hydrolysate when compared to the one used for the original adaptation. Specifically, we analyzed transcript levels arising from genes adjacent to SNPs identified in strain SS3 that differed from the parental strain 8b: ZMO0152, ZMO0153, ZMO0774, ZMO0776, ZMO0959, and ZMO0961. We further analyzed transcript levels of several genes within the glycolytic pathway [ZM01360 (*pdc*), ZMO1596 (*adhB*), and ZMO1608 (*eno*)]. The full results can be seen in Table 2. Interestingly, we identified two genes whose transcripts were significantly reduced (twofold or more and a paired T-test *P* ≤ 0.05) in strain SS3 compared to strain 8b in both rich medium and hydrolysate conditions. Specifically, ZMO0153 and ZM00776 transcripts were reduced near fivefold in strain SS3 relative to strain 8b in rich medium (*P* = 0.0076 and *P* = 0.003, respectively) and near twofold in hydrolysate (*P* = 0.012 and *P* = 0.015, respectively). While functionally uncharacterized, ZMO0153 is annotated based on homology as a potential transcriptional regulator (UniProt accession number Q5NR77) [18]. ZMO0776 encodes a predicted type 12 methyltransferase which is closely related to an annotated phospholipid N-methyltransferase in *Z. mobilis* strain CP4 (Genbank Accession AHB09844); however, no physical characterization of this gene product has been published to our knowledge.

**Table 1:** **Compositional analysis of saccharified pretreated corn stover hydrolysates used for adaptation and evaluation**

	**Cellobiose**	**Glucose**	**Xylose**	**Galactose**	**Arabinose**	**Acetic acid**	**HMF**	**Furfural**
	**g/L**	**g/L**	**g/L**	**g/L**	**g/L**	**g/L**	**g/L**	**g/L**
Material used for adaptation								
As is (P050921CS)	3.35	74.17	46.46	4.14	7.69	10	2.1	1.8
Material used for evaluation								
De-acetylated (P120927DCS)	3.78	99.98	51.03	2.94	7.79	2.2	0.14	0.91

**Table 2:** **Differential transcript levels between 8b and SS3**

		**Gene flanking SNP changes**						**Genes in glycolysis and fermentation pathway**		
		**ZMO 0152**	**ZMO 0153**	**ZMO 0774**	**ZMO 0776**	**ZMO 0959**	**ZMO 0961**	**ZMO 1360 (pdc)**	**ZMO 1596 (adhB)**	**ZMO 1608 (eno)**
Pure glucose	8b	3.63 ± 0.02	5.45 ± 0.26	3.34 ± 0.02	4.52 ± 0.15	3.73 ± 0.11	3.73 ± 0.19	5.15 ± 0.13	5.98 ± 0.27	5.05 ± 0.49
	#3	3.69 ± 0.17	4.74 ± 0.15	3.47 ± 0.11	3.88 ± 0.28	3.69 ± 0.12	3.54 ± 0.06	5.23 ± 0.07	6.08 ± 0.01	5.13 ± 0.02
	Ratio (#3/8b)	1.14	0.19	1.36	0.23	0.92	0.64	1.21	1.25	1.2
	T-test	0.34	0.0076	0.057	0.012	0.35	0.085	0.2	0.28	0.4
40% DCS	8b	3.68 ± 0.11	5.35 ± 0.03	3.43 ± 0.20	4.41 ± 0.10	3.76 ± 0.23	3.75 ± 0.06	5.34 ± 0.02	5.82 ± 0.05	4.87 ± 0.11
	#3	3.63 ± 0.16	5.06 ± 0.09	3.21 ± 0.05	4.17 ± 0.07	3.64 ± 0.12	3.53 ± 0.13	5.16 ± 0.15	5.96 ± 0.05	4.99 ± 0.12
	Ratio (#3/8b)	0.88	0.51	0.61	0.58	0.75	0.6	0.66	1.387	1.31
	T-test	0.33	0.003	0.071	0.015	0.22	0.025	0.056	0.012	0.14
8b	Ratio (DCS/)	1.14	0.8	1.23	0.77	1.07	1.04	1.54	0.7	0.66
	T-test	0.28	0.28	0.23	0.17	0.42	0.44	0.036	0.19	0.28
#3	Ratio (DCS/)	0.88	2.13	0.56	1.94	0.88	0.97	0.84	0.77	0.71
	T-test	0.35	0.015	0.011	0.078	0.3	0.43	0.24	0.009	0.052

Transcript quantification is shown (Log_10_) for the indicated genes in either rich medium or in 40% de-acetylated corn stover hydrolysate. SS3:8b transcript ratios are shown, with the paired T-test values indicated below. Results highlighted in blue represent statistically significant values (*P* ≤ 0.05), and results highlighted in bold and blue represent statistically significant values with differential values near twofold or above.

In addition to comparing transcript levels directly between strains 8b and SS3, we also wanted to determine if these genes were expressed differentially in rich medium as compared to in hydrolysate. Overall, it appeared that there was very little difference in the transcripts we measured whether cells were grown in rich medium with glucose or in 40% deacetylated corn stover hydrolysate. However, two exceptions to this trend were observed in genes ZM0153 and ZM0774 in strain SS3. ZMO0153 was upregulated approximately twofold (*P* = 0.015), and ZMO0774 was reduced approximately twofold (*P* = 0.011). This media-dependent change was not observed in strain 8b, suggesting that this differential expression is unique to strain SS3. Neither the mechanism nor the consequences of this altered gene expression is understood at this point, and overall this study raises several questions for which follow-up research will be necessary.

Additional research will be required to determine if ZMO0153 truly encodes a functional transcription factor and what targets are up- or downregulated in response to its downregulation in SS3 as shown here. Similarly, in-depth characterization of ZMO0774 and ZMO0776 may help shed light on their role in the enhanced performance of strain SS3. Differential global transcriptomic analysis between SS3 and 8b or ChIP-Seq methods may provide deeper insights into transcriptional targets and the effects of altered expression of these genes.

## Conclusion

We have successfully adapted/evolved strain 8b to consume more xylose and produce more ethanol in a toxic dilute-acid hydrolysate, ultimately generating strain SS3. Ethanol yields were 18% higher, and xylose utilization rates were 59% faster in strain SS3 as compared to parental strain 8b. Additionally, we have shown that SS3 maintains higher viability on a xylose-containing medium following fermentation, suggesting a higher level of fitness in this strain during hydrolysate fermentation. To begin to understand the genotype behind this improved phenotype, we used NGS technologies to sequence the genome of SS3. We have identified several SNPs that may be responsible for this phenotype. Among the genes flanking the SNPs, we learned that two genes encoding a putative transcription factor and a family 12 methyltransferase were significantly downregulated greater than twofold in SS3 background compared to parental strain 8b. Additional studies will be required to understand the fundamental mechanism behind these genes and their contribution towards the enhanced productivity of strain SS3. While the fundamental genetic and metabolic model remains somewhat unclear, what is clear is that we have successfully generated a strain through adaptive evolution that can more efficiently metabolize xylose and generate higher ethanol titers than the parental strain, and that this strain offers novel insights into further hypothesis-based strain improvement strategies.

## Materials and methods

### Microorganism and culture media

Strain *Z. mobilis* 8b [1] was used as the parent strain for adaptation and improvement during the course of this work. The culture media used were rich media (RM) composed of 10 g/L yeast extract and 2 g/L potassium phosphate, prepared as 10X stock solution supplemented with desired sugar.

### Pretreated corn stover (PCS) for whole slurry fermentation

Corn stover 34 M95 was pretreated in a pilot-scale 1 ton/day vertical reactor at a solids loading of 30% (w/w), temperature of 190°C, and 0.048 g sulfuric acid per gram dry biomass with 1-min residence time. The hydrolysate was neutralized with 30% ammonium hydroxide from pH 1.8 to 5.8 and then filter sterilized. For whole slurry fermentation, 25% total solids (TS) was neutralized using 30% ammonium hydroxide to pH 5.1; then it was enzymatically hydrolyzed. For gene expression experiments, de-acetylated saccharified liquor was used due to unavailability of original liquor. De-acetylation was performed at 8% (w/w) total solids (TS) concentration with 1,500 kg total mass at 80°C, 2 h, and 0.4% (w/w) NaOH in the NREL Dynamic Impregnator (DI) vessel. The DI was mixed at 15 rpm during de-acetylation. After de-acetylation, the spent caustic liquor (SCL) was drained from the vessel, leaving the remaining solids at 12% TS. The remaining solids were then rinsed with 950 kg of water, which was drained from the vessel and discarded [17].

### Enzymatic hydrolysis of pH-adjusted PCS

The pH-adjusted PCS slurry was enzymatically hydrolyzed with Cellic CTec cellulase enzyme (Novozymes, Davis, CA, USA). The enzyme was added at 40 mg protein/g cellulose and incubated at 48°C and 200 rpm for 4 days. When hydrolysis was complete, we transferred the saccharified PCS slurry into the bioreactors for fermentation.

### Continuous adaptation to select hydrolysate-tolerant strains

A long-term (70-day) continuous fermentation was used to adapt the *Z. mobilis* strain 8b to ammonia-neutralized PCS. *Z. mobilis* strain 8b was inoculated into a 100-mL working volume fermentor with rich media (RM), 100 g/L glucose (G), and 20 g/L xylose (X)-designated RMGX (10%:2%), with the levels (w/v) of glucose and xylose in parentheses. The pH of the batch fermentation was controlled at pH 5.8 and temperature 33°C. When glucose was consumed to 40 g/L, 25% diluted neutralized hydrolysate liquor was fed at a dilution rate of 0.03 to 0.05 h^−1^. When a steady state was reached, the hydrolysate level was increased in 5% increments up to a level of 60%. The turbidostat was stopped when the cell concentration dropped below 10^3^ cells/mL and glucose began to accumulate (60% hydrolysate level). Before stopping the run, a sample of the culture was frozen with 25% glycerol (final concentration) and also plated on RMGHyd (rich media supplemented with glucose and hydrolysate) (2% glucose:40% hydrolysate). Twenty colonies were characterized on the hydrolysate plate, and one had better performance and was selected and termed SS3 (sub-strain 3) for further evaluation.

### Fermentation evaluation of adapted strains

*Zymomonas mobilis* 8b and SS3 were taken from cell stocks stored at −70°C. The pre-seed medium consisted of 1X RM, supplemented with 80 g/L glucose and 20 g/L xylose. The culture was revived by transferring 1 mL of the frozen stock into 9 mL of pre-seed medium in a 15-mL tube. The cultures were incubated at 33°C with no agitation and then sampled at 8 h for an optical density reading at 600 nm using a Spectronic 601 spectrophotometer (Milton Roy, Ivyland, PA, USA). The pre-seed culture was used to inoculate the batch seed fermentor, if needed, with media composition of RM (1X), 150 g/L glucose, 20 g/L xylose, and 1 g/L sorbitol. Temperature was controlled at 33°C and pH at 5.8 by 4 M KOH.

Fermentations to evaluate the strains were done in BioStat-Q plus fermenters with a 300 mL working volume, 300 rpm, a temperature of 33°C, and pH 5.8 controlled with KOH (2 N). Saccharified liquor or hydrolysate used in fermentation was enriched with RM. The fermenters were inoculated with an OD 600 nm value of 1 using a direct transfer procedure (10% v/v). Ethanol yield was calculated based on initial sugar concentrations and differences between final and initial ethanol concentration.

### Fermentation, data collection, and analysis

Samples from the fermentations were taken at various time points. The samples were centrifuged and supernatants were filtered through a 0.2-μm syringe filter before placing in high-pressure liquid chromatography (HPLC) vials. The samples were then analyzed for carbohydrates and organic acids [19]. Analysis on the carbohydrate HPLC was performed using the Shodex SP0810 carbohydrate column, and organic acids analysis was performed using the Bio-Rad Aminex HPX-87H organic acids column. Sugar utilization, ethanol yield, and ethanol titers were calculated based on this HPLC data. We also determined the insoluble solids in the slurry and the density of the liquor for the initial and final time points.

### Genomic re-sequencing analysis of SS3 through next-generation sequencing (NGS)

Genomic DNA was extracted using Qiagen DNA Easy Kit (Qiagen, Valencia, CA, USA) and sent to the University of Utah for next-generation sequencing using Illumina Hiseq2000 after passing the quality requirements. The quality of FASTQ genome re-sequencing data delivered from the University of Utah was checked using the FastQC program; data passing the quality control were imported into the CLC Genomics Workbench 5.5.1 for genome mapping and resequencing analyses to identify the potential small genetic changes such as SNP and indels in the mutant strain.

### Fermentation for qRT-PCR experiment and qRT-PCR

Fermentations to generate cell samples for the qRT-PCR were performed in 125-mL shake flasks with 75 mL of culture media. Both strains 8b and SS3 were grown on 50% de-acetylated saccharified PCS liquor supplemented with RM media. The flasks were incubated at 30°C shaker at 180 rpm. Samples were taken at mid-log phase and centrifuged at 8,000 rpm (Sorvall LYNX 6000, Thermo Scientific, Waltham, MA, USA) for 10 min. The supernatants were discarded, and the cells were immediately frozen using liquid nitrogen and stored at −80°C until ready for analysis.

RNA extraction and quantification, cDNA synthesis, and real-time qPCR were performed as described previously (6, 20 to 24), except that the Bio-Rad CFX96 Touch™ Real-Time PCR Detection System (Bio-Rad Lab, Hercules, CA, USA) and SsoFast™ EvaGreen® Supermix (Bio-Rad Lab, CA) were used for this study. Briefly, total cellular RNA was extracted using the TRIzol reagent (Invitrogen, Carlsbad, CA, USA) followed by RNase-free DNase I (Ambion, Austin, TX, USA) digestion. RNA quality and quantity were tested with a NanoDrop ND-1000 spectrophotometer (NanoDrop Technologies, Wilmington, DE, USA) and Agilent Bioanalyzer (Agilent, San Diego, CA, USA). Purified RNA of high quality was used as the template to generate ds-cDNA using iScript™ Advanced cDNA Synthesis Kit for RT-qPCR kit (Bio-Rad Lab, CA). The expression levels of nine genes flanking the SNP changes or involved in glycolysis pathway were analyzed using RT-qPCR. The expression level was based on the copy number of each gene in either RMG8 or hydrolysate condition using the absolute quantification approach as described previously [6,20-24]. Primer pairs were designed using Primer3 webserver [25], and the oligonucleotide sequences of the nine genes selected for qPCR analysis are listed in Table 3.

**Table 3:** **Primers used for the qRT-PCR experiment reported in Table** 2

**Primer_ID**	**Primer sequence**
*ZMO0774_qF2*	CGCTCGATATTCTGGCTGAG
*ZMO0774_qR2*	TTTTGTGAGGCGTAAAGGGG
*ZMO0776_qF2*	ACCGGATGCGACCTTGAT
*ZMO0776_qR2*	AGACCCATTCACGGCGATAA
*ZMO0959_qF2*	AACAGGCGGCCAAACATAAC
*ZMO0959_qR2*	CCGCCATTTTGCCATAGAAA
*ZMO0961_qF2*	AGAATATGCCGCAGATCAAGC
*ZMO0961_qR2*	TTTTGCCGTTCGATCGTCAT
*ZMO0152_qF1*	TCGATCAGGACCCAACTCTC
*ZMO0152_qR1*	TCATCCAAAAGCAGGCGATG
*ZMO0153_qF2*	GAGCATGAAATCTGGACCGC
*ZMO0153_qR2*	ACGCCATGCCAATTTCACG
*ZMO1608_qF1*	CTGGAAGCCGAAGATCAGGA
*ZMO1608_qR1*	TGACACCGAGGATAGCGTTA
*ZMO1360_qF1*	CGGTTTCAGTGCAGAAGGTT
*ZMO1360_qR1*	GCCACCGATAGCATCAAATG
*ZMO1596_qF2*	AATCAACACGACGGCTGGTA
*ZMO1596_qR2*	AACGTGACGGTCAACAATGG

### Microscopic examination of strains

Fermentation samples were examined by light microscopy (Nikon eclipse E800, Tokyo, Japan). Representative micrographs were captured at × 1000 magnification as shown in Figure 4.

## Abbreviations

HMF: hydroxymethylfurfural
NGS: next-generation Sequencing
PCS: pretreated Corn Stover
qRT-PCR: reverse transcription PCR
RM: rich media
RMG: rich media supplemented with Glucose
RMGHyd: RMG supplemented with hydrolysate
SS3: sub-strain 3
TS: total solids
V/V: volume per volume
W/W: weight per weight
SCL: spent caustic liquor
